# A Population-Based Case-Control Study of Drinking-Water Nitrate and Congenital Anomalies Using Geographic Information Systems (GIS) to Develop Individual-Level Exposure Estimates

**DOI:** 10.3390/ijerph110201803

**Published:** 2014-02-05

**Authors:** Caitlin E. Holtby, Judith R. Guernsey, Alexander C. Allen, John A. VanLeeuwen, Victoria M. Allen, Robert J. Gordon

**Affiliations:** 1 Department of Community Health and Epidemiology, Dalhousie University, 5790 University Avenue, Halifax, NS B3H 1V7, Canada; E-Mails: jrg@dal.ca (J.R.G.); victoria.allen@dal.ca (V.M.A.); 2 Perinatal Epidemiology Research Unit, Departments of Pediatrics and Obstetrics & Gynaecology, Dalhousie University, 5980 University Avenue, Halifax, NS B3K 6R8, Canada; E-Mail: alexander.allen@dal.ca; 3 Department of Health Management, Atlantic Veterinary College, University of Prince Edward Island, 550 University Avenue, Charlottetown, PE C1A 4P3, Canada; E-Mail: jvanleeuwen@upei.ca; 4 Department of Obstetrics and Gynaecology, Dalhousie University, 5980 University Avenue, Halifax, NS B3K 6R8, Canada; 5 School of Environmental Sciences, University of Guelph, Guelph, ON N1G 2W1, Canada; E-Mail: rjgordon@uoguelph.ca

**Keywords:** Nitrate, Congenital anomalies, Drinking-water, Geographic Information Systems (GIS)

## Abstract

Animal studies and epidemiological evidence suggest an association between prenatal exposure to drinking water with elevated nitrate (NO_3_-N) concentrations and incidence of congenital anomalies. This study used Geographic Information Systems (GIS) to derive individual-level prenatal drinking-water nitrate exposure estimates from measured nitrate concentrations from 140 temporally monitored private wells and 6 municipal water supplies. Cases of major congenital anomalies in Kings County, Nova Scotia, Canada, between 1988 and 2006 were selected from province-wide population-based perinatal surveillance databases and matched to controls from the same databases. Unconditional multivariable logistic regression was performed to test for an association between drinking-water nitrate exposure and congenital anomalies after adjusting for clinically relevant risk factors. Employing all nitrate data there was a trend toward increased risk of congenital anomalies for increased nitrate exposure levels though this was not statistically significant. After stratification of the data by conception before or after folic acid supplementation, an increased risk of congenital anomalies for nitrate exposure of 1.5–5.56 mg/L (2.44; 1.05–5.66) and a trend toward increased risk for >5.56 mg/L (2.25; 0.92–5.52) was found. Though the study is likely underpowered, these results suggest that drinking-water nitrate exposure may contribute to increased risk of congenital anomalies at levels below the current Canadian maximum allowable concentration.

## 1. Introduction

Congenital anomalies complicate 2% to 3% of Canadian births and the incidence of neural tube defects, congenital heart defects and Down’s syndrome in Nova Scotia are among the highest in Canada [1]. The etiologies of many congenital anomalies remain unknown; their widespread health impacts warrant further investigation into risk factors, environmental causes and means of prevention [1]. 

Teratogens in the environment, such as nitrate, may cause 8%–12% of congenital anomalies [1,2]. Nitrate (NO_3_) is one of the most prevalent forms of biologically-available nitrogen, and may be created naturally or anthropogenically [3,4,5]. Nitrate is soluble in water and has the ability to leach into groundwater [3,4,5].

Ingested nitrate may be converted to nitrite by microbial reduction in saliva, or in the stomach during instances of increased pH or infections with diarrhea-producing bacteria [6,7]. Nitrites can react with amines and other nitrosatable compounds to produce highly reactive N-nitroso compounds in the stomach [6,7]. Several animal studies have shown that nitrates and other nitrogenous compounds can cross the placenta and have a teratogenic effect on the developing fetus during pregnancy, particularly impacting the central nervous system [8,9,10]. Several case-control studies have shown a positive association between drinking-water nitrate levels and incidence of congenital anomalies among humans [11,12,13,14,15]. Dorsch *et al.* showed increased risk of all congenital anomalies for nitrate concentrations >5 mg/L [11]. Arbuckle *et al.* showed an increased risk of central nervous system (CNS) anomalies in New Brunswick, Canada, for nitrate concentrations >26 mg/L and Croen *et al.* showed an increased risk of anencephaly for those whose mothers drank groundwater with nitrate concentrations >6.9 mg/L [12,13]. Studies by Cedergren *et al.* and Manassaram *et al.* showed trends toward associations between drinking-water nitrate and anomalies of the cardiac and central nervous systems respectively [14,15]. 

This study was designed to estimate the association between drinking-water nitrate concentrations and incidence of major congenital anomalies in the agricultural region of Kings County, Nova Scotia, Canada. It expanded on existing research in four main ways: (1) the use of province-wide population-based databases enabled controlling for a number of health and demographic variables that may confound the association between drinking-water nitrate and congenital anomalies; (2) we were able to include cases diagnosed *in-utero* with congenital anomalies in pregnancies that were electively terminated before birth; (3) we included users of both private wells and municipal water supplies in the study population and; (4) individual-level nitrate exposure estimates were derived from temporally monitored well data with fine geographic resolution, using Geographic Information Systems (GIS), providing confidence in these estimates.

## 2. Experimental Section

### 2.1. Research Ethics Board Approvals

To ensure confidentiality, the Reproductive Care Program of Nova Scotia selected cases and controls using the selection criteria described below. Approval for access to data in the Nova Scotia Atlee Perinatal Database (NSAPD) was granted by the Data Access Committee of the Perinatal Epidemiology Research Unit, the Dalhousie University Population Health Research Unit, and the Reproductive Care Program of Nova Scotia. Approval for the use of data in the Dalhousie University Department of Obstetrics and Gynecology Fetal Anomaly Database (FAD) was granted by the FAD Data Access Committee. The project received ethical approval from the IWK Health Centre Research Ethics Board and the Annapolis District Health Authority Research Ethics Board. 

### 2.2. Study Area

The study was undertaken in Kings County (total 2,001 population = 58,866) located in the predominantly agricultural Annapolis Valley, in South-Western Nova Scotia, a province in Eastern Canada. Kings County was selected because a series of wells along the intensively-farmed valley floor have had routine nitrate concentrations measured repeatedly in 1989, 1999 and 2000 to examine seasonal and temporal variations. Water quality in Kings County is usually good, though it is susceptible to contamination from surficial sources along the valley floor [10,16,17]. In 1999, 44% of the 140 wells sampled in the study area had nitrate-nitrogen concentrations (hereafter referred to as nitrate concentration) above 5 mg/L, and 19% had nitrate concentrations above the 10 mg/L Health Canada Maximum Allowable Concentration (MAC) [10]. Kings County includes the municipalities of Canning, Wolfville, Kentville, New Minas, and Port Williams. Each municipality has its own water supply described in Table 1 [18,19,20,21]. 

**Table 1:** Descriptive statistics of nitrate-nitrogen concentrations (mg/L) for all water sources in each municipal water supply from 1996 to 2003 in comparison to all sampled rural wells in Kings County from 1999–2000. Nitrate exposure level refers to the nitrate concentration categorization used in subsequent analyses to determine the association between congenital anomalies and nitrate exposure.

Location	Water Source	# of Sample Locations	Total # of Samples	Min. [Nitrate]	Max. [Nitrate]	Mean [Nitrate] *	Median [Nitrate)	Nitrate Exposure Level
All rural wells	Groundwater	140	1,113	0.0	43.0	6.44 (5.99–6.89)	3.8	n/a
All municipalities	Ground and surface water	20	53	0.0	10.4	2.03 (1.58–2.48)	1.7	n/a
Canning	Groundwater	2	24	1.0	2.5	1.63 (1.47–1.80)	1.7	1.0–5.56
Greenwood	Groundwater	2	5	0.0	1.0	0.63 (0.12–1.14)	0.9	<1.0
Kentville	Surfacewater	1	2	0.4	1.0	0.69 (0.00–4.28)	0.7	<1.0
New Minas	Groundwater	9	10	1.0	2.9	1.68 (1.92–2.07)	1.6	1.0–5.56
Port Williams	Groundwater	4	6	1.4	10.4	5.08 (1.64–8.52)	4.6	1.0–5.56
Wolfville	Groundwater	2	6	2.4	3.6	2.77 (2.33–3.21)	2.7	1.0–5.56

Note: * 95% CI.

### 2.3. Data Sources

All cases and controls were selected from either the NSAPD or FAD. The NSAPD was established in 1988 and is a province-wide population-based birth registry database. The NSAPD contains systematically-recorded information on maternal and infant demographics, as well as information on medical procedures, interventions, diagnoses (including congenital anomalies) and health outcomes for all births in Nova Scotia. This information is recorded through a formal coding process at a central location. Data are obtained from hospital records, physician reports, prenatal diagnostic facilities, cytogenetic laboratories, maternal serum screening programs and vital statistics. The FAD was established in 1992 and records information on all Nova Scotia fetal anomalies diagnosed during pregnancy, including those pregnancies that underwent second trimester terminations. FAD data are obtained in similar fashion as is done for the NSAPD. Linkages between the NSAPD and the FAD are made using Nova Scotia provincial health card numbers.

Latitude and longitude of maternal addresses were determined using the Nova Scotia Civic Address File, which was introduced province-wide in 1998 to support the implementation of the provincial Emergency Health Service. The latitude and longitude of the centre-point of every dwelling in Nova Scotia has been measured using a handheld GPS unit, resulting in location estimates that are accurate within 2.5 m [22], and recorded in a central spatial database for use in response to 911 emergency calls.

### 2.4. Inclusion and Exclusion Criteria

All fetuses or infants diagnosed with a major congenital anomaly between 1 January 1988 and 31 December 2006 with a maternal residential address within the Kings County boundaries were selected as cases. Infants born without a major congenital anomaly over the same time period, also with a maternal residential address within the Kings County boundaries were selected as controls. Cases and controls were matched at a 1:3 ratio based on infant’s sex and date of conception (within +/− 30 days) to enhance statistical power. For all cases and controls, the maternal address had to be available and in Kings County at the time of delivery. Twins and higher-order multiple births were excluded from the study. Principally an agricultural region, residential mobility is low in Kings County compared to Nova Scotia as a whole.

### 2.5. Demographic and Health Data Collection

In addition to the sex of the infant and season of conception (Spring: 20 March–20 June; Summer: 21 June–21 September; Fall: 22 September–20 December; Winter: 21 December–19 March), maternal demographic variables and information regarding maternal risk factors for congenital anomalies was obtained from the NSAPD and the FAD. These include: maternal age at conception, maternal parity (defined as the number of times a woman had given birth to an infant or stillbirth having a gestational age of 20 weeks or more, or having a birthweight of 500 grams or more), smoking (either pre-pregnancy or at first prenatal visit), pre-existing or gestational diabetes, pre-existing thyroid disease, patient-reported folate supplementation at the time of conception, patient-reported pre-pregnancy weight, and conception before or after folate fortification in Nova Scotia (determined by month and year of conception). 

### 2.6. Nitrate Concentration Measurement

**Figure 1:**
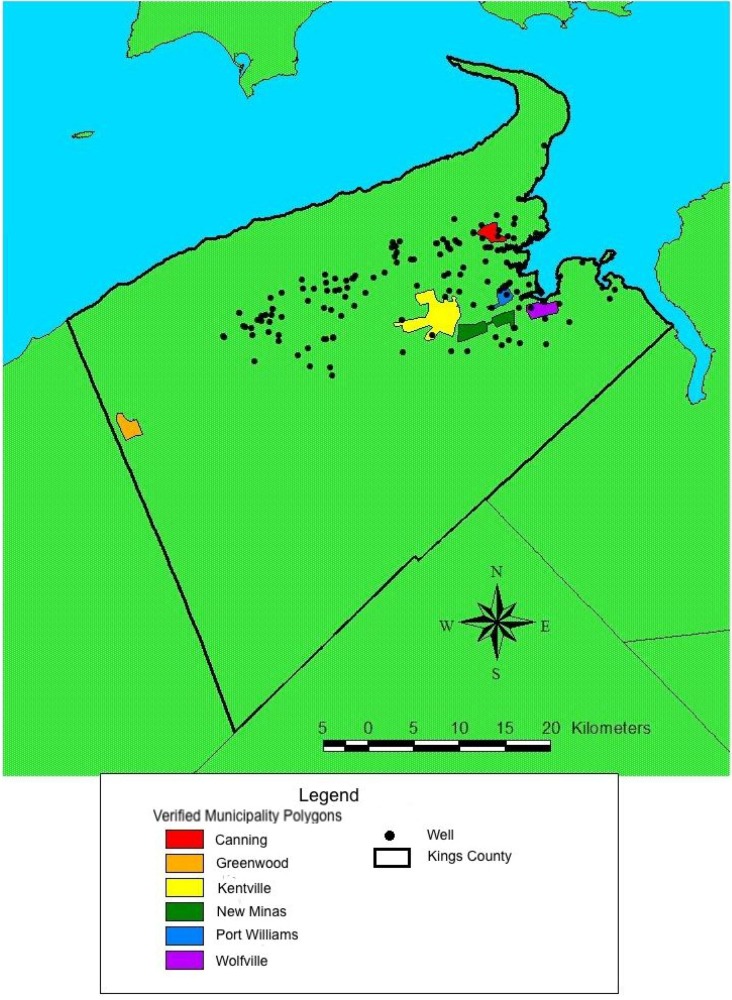
Map of Kings County, Nova Scotia, highlighting the six small town municipal water supplies and the locations of the rural wells from which nitrate concentrations were monitored in 1999 and 2000.

For study participants residing in regions served by municipal water supplies, nitrate exposure estimates were based on water system nitrate concentrations measured between 1996 and 2003 provided by each of the six water supply plants (Figure 1). At these plants, water samples were taken with variable frequencies and at irregular intervals over the study period. For the municipal water supplies as an aggregate, analyses were conducted to evaluate the variation in nitrate concentration over time in each municipality. Linear mixed effects models were used to assess the effects of location of the sample (if samples were drawn from multiple locations within a single municipality), month of sample, and year of sample on log-transformed nitrate concentrations. 

**Figure 2:**
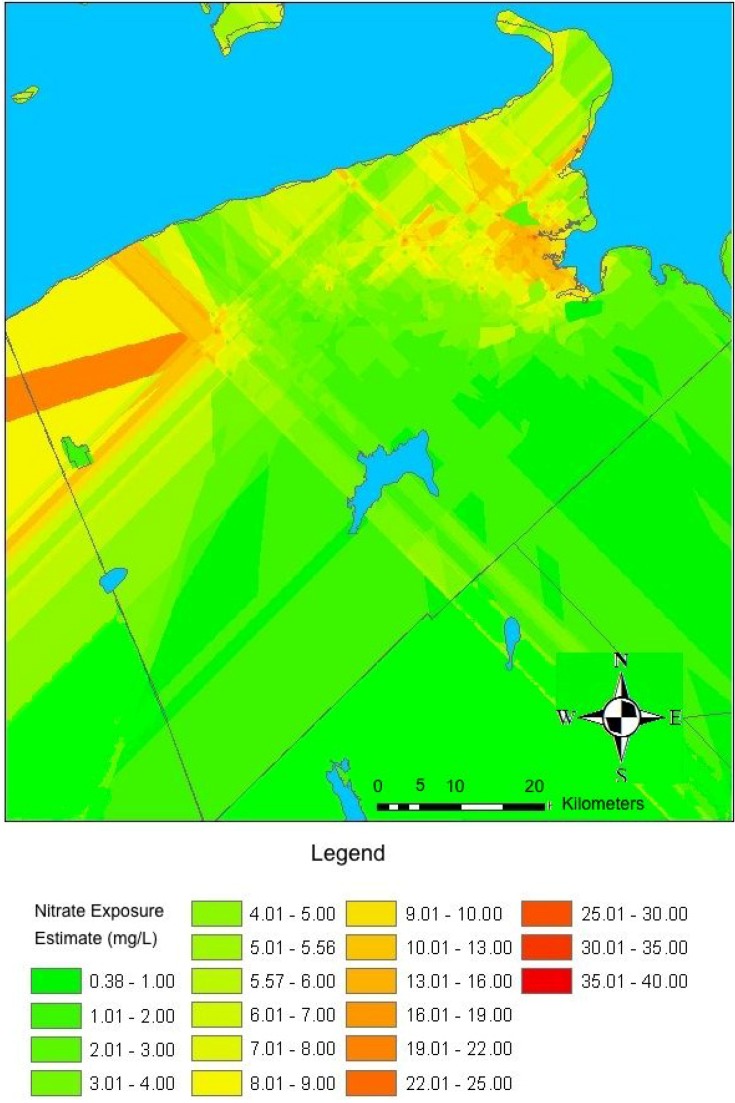
Map showing the drinking-water nitrate exposure estimates for Kings County, Nova Scotia, that were derived using ordinary kriging and extrapolation estimates in rural areas and statistically derived estimates of average nitrate concentrations in municipal water supplied regions.

Sample location was considered a random effect, while month of sample and year of sample were considered fixed effects. Similar methods have previously been used to assess temporal changes in groundwater nitrate measurements [23,24]. Year and month of the water samples from the municipal water supplies were not significantly associated with nitrate concentration (Year: *p* = 0.92, Month: *p* = 0.38). Sample location alone described most of the variation in nitrate levels (R^2^ = 0.74, *p* < 0.01). Therefore, the median of all nitrate concentration measurements taken within each municipal water supply was used as the nitrate exposure estimate for all study participants living in each municipality (Figure 2).

In rural areas, drinking-water nitrate concentrations were estimated using GIS (see below) from the nitrate concentrations of monthly samples taken from 140 wells in 1999 and 2000. These wells were among 237 originally selected for routine monitoring by the Nova Scotia Department of Environment and Labour and the Nova Scotia Agricultural College according to the DRASTIC model in 1989. The DRASTIC model integrated information regarding the location of various agricultural crops and soils to estimate susceptibility to nitrate contamination beyond the natural background level [25,26]. Wells were selected such that they represented a range of susceptibilities toward nitrate contamination [25]. Monitoring took place monthly from July 1999 to February 2000. Linear mixed effects modeling was used to evaluate the variation in nitrate concentrations between wells, as well as variation in nitrate concentration of the water from each well for different months and years. The year and month in which the water samples were taken were not associated with nitrate concentration (Year: *p* = 0.61; Month: *p* = 0.12) and well location described most of the variation in nitrate in rural areas (R^2^ = 0.74, *p* < 0.01). Therefore, a single map representing rural nitrate concentration estimates based on location of wells was created.

### 2.7. Exposure Assessment

The exposure assessment for participants in our study was conducted using ArcGIS software (ESRI, Redlands, CA, USA). First, ordinary kriging was used to estimate nitrate concentrations in areas near the 140 monitored wells. The final spatial resolution of the derived raster surface was 1:2.2 with a cell size of 221 m. This model was then extrapolated to cover all of Kings County. For details of the kriging and extrapolation process used to estimate nitrate exposure in rural areas see the supplementary material. All interpolation models considered, as well as their parameters and associated errors are included in Table S1. Next, maps delineating the service boundaries of each municipal water supply were used to draw freehand polygons representing the geographic range of each water supply distribution system in ArcGIS^TM^. Lists of addresses served by each municipality were used to confirm the accuracy of the polygons, which were cross-checked by each water supply manager. Next, the regions served by municipal water supplies were “cut-out” from the map representing rural nitrate concentrations and the median value of all nitrate measurements for each municipal water supply was imputed into the appropriate polygon. The latitude and longitude of the maternal address at the time of delivery was then used to determine a nitrate-exposure estimate for each study participant. 

The nitrate concentration estimates (represented as mg nitrate-nitrogen) were divided into drinking-water nitrate exposure level categories (<1 mg/L, 1–5.56 mg/L, >5.56 mg/L), with 1 mg/L demarcating pristine areas from anthropogenically-affected areas in Atlantic Canada and 5.56 mg/L consistent with the European guideline for a “safe” level of nitrate in drinking-water [27,28].

### 2.8. Statistical Analyses

Frequency tables and univariable analyses using unconditional logistic regression were created for all covariates included in the study (infant sex, season of conception, year of conception, maternal age, maternal parity, pre-pregnancy weight, smoking, thyroid disease, diabetes, folate supplementation, folate fortification, ground *vs.* surface water, municipal *vs.* private water supply, and nitrate exposure). A step-wise multivariable unconditional logistic regression, including those variables on which the study participants were matched, was used to estimate the odds ratios (OR) and 95% confidence intervals (95% CI) for the incidence of congenital anomalies by drinking-water nitrate exposure level. Since study participants were matched on very few parameters relative to the total sample size, unconditional logistic regression was used to examine associations in this study.

Covariates were added to the multivariable model by group in a stepwise fashion in order to assess their impacts on the association between drinking-water nitrate and congenital anomalies. Groups of covariates were added to the model in the following order: (1) matching variables (sex, season of conception, year of conception); (2) demographic variables (maternal age, parity); (3) maternal risk factors (smoking, diabetes, thyroid disease); (4) water source variables (surface *vs.* groundwater, municipal *vs.* rural) and finally; (5) nitrate exposure level. This process enables stepwise comparison of the impact of the addition of each set of covariates to the overall point estimates and confidence intervals for previously introduced parameters. The final column shows the results from the nitrate exposure levels after all previous variables of interest are accounted for.

The logistic regression analyses were repeated after stratifying the data by date of conception before or after 1 January 1998. The year 1998 was chosen as the year of stratification because it is the year in which Canada first began to fortify food containing grain with folic acid nationally for the purpose of ensuring pregnant women were receiving sufficient quantities in their diets. It also coincides with the introduction of civic geocoding (exact latitude and longitude) of all addresses of all homes in Nova Scotia thereby enhancing accuracy of geographic location where the estimated exposures occurred. 

## 3. Results and Discussion

### 3.1. Results

Descriptive statistics for the nitrate measurements for the rural wells in aggregate, as well as all six municipal water supplies, are shown in Table 1. The mean and median drinking-water nitrate levels were below the MAC in both municipalities and rural areas, though the mean, median and maximum drinking-water nitrate concentrations were higher in rural areas compared to those within municipalities. Some nitrate measurements from rural wells, as well as at least one from the municipality of Port Williams, were above the MAC.

Frequency of congenital anomalies by body system for the entire study period, as well as stratified for before and after 1998, are presented in Table 2. Congenital anomalies of the central nervous system were the most common throughout the entire study period. 

**Table 2:** Frequency of diagnoses of major congenital anomalies in Kings County, categorized by body system, over the entire study period (1987–2006), during the period prior to the fortification of food in Canada with folate (1987–1997), and for the period after folate fortification (1998–2006) *.

Class of Anomaly	Entire Study Period		Before Folate Fortification		After Folate Fortification	
	1987–2006		1987–997		1998–2006	
	Number of cases	Percentage of all congenital anomalies	Number of cases	Percentage of all congenital anomalies for period	Number of cases	Percentage of all congenital anomalies for period
All Anomalies	606	100	300	100	306	100
Central Nervous System	286	47	98	33	188	61
Musculoskeletal System	101	17	58	19	43	14
Genitourinary System	50	8	25	8	25	8
Cardiovascular System	47	8	26	9	21	7
Inguinal Canal	37	6	26	9	11	4
Multiple Anomalies	33	5	24	8	9	3
Eye, Ear, Nose, Throat and Mouth	27	4	17	6	10	3
Other	27	3	16	5	11	4
Missing	53	9	53	18	0	0

Note: * All categories containing fewer than five cases were amalgamated.

Frequency tables and odds ratios comparing cases to controls for covariates, as well as nitrate exposure estimates are summarized in Table 3. There was an increased likelihood of congenital anomalies with maternal smoking and a protective effect shown for maternal parity of 1–2. Provision of water from a private or municipal water supply, ground or surface water source, and drinking-water nitrate exposure level did not differ between cases and controls. 

**Table 3:** Distribution of matching, demographic, health and water source variables, including nitrate exposure level, between cases of congenital anomalies and controls, as well as univariable logistic regression odds ratios between incidence of congenital anomalies and all variables for cases and controls (1987–2006).

Variable	Cases (n = 606)		Controls (n = 1,635)		Crude Odds Ratio **	*p*-value *
	n	%	n	%		
Sex						0.91
Female	268	48	788	48	1.0	
Male	285	52	847	52	0.99 (0.82–1.20)	
Missing	53		0			
Season of conception						0.96
Winter	83	14	244	15	1.0	
Spring	139	15	393	24	1.04 (0.76–1.43)	0.63
Summer	162	25	484	30	0.98 (0.73–1.34)	0.87
Fall	169	29	514	31	0.97 (0.71–1.31)	0.70
Missing	53		0			
Year of Conception						
1987–1991	83	15	245	15	1.0	
1992–1996	113	20	342	21	0.98 (0.70–1.35)	0.75
1997–2001	282	51	838	51	0.99 (0.75–1.32)	0.87
2002–2006	75	14	210	13	1.05 (0.73–1.52)	0.67
Missing	53		0			
Parity						**0.01**
0	273	49	687	42	1.0	
1–2	252	46	852	52	**0.74 (0.61–0.91)**	0.29
3+	28	5	96	6	0.73 (0.47–1.14)	0.46
Missing	53		0			
Pre-pregnancy weight (kg)						0.55
<50						
50–69	44	9	129	9	1.02 (0.70–1.47)	0.68
≥70	281	59	837	56	1.0	
Missing	155	32	520	35	0.89 (0.71–1.11)	0.33
	126		149			
Smoker						
No	358	59	1140	70	1.0	
Yes	248	41	495	30	**1.60 (1.32–1.94)**	**<0.001**
Thyroid						
No	604		1,626	99	1.0	
Yes	2	100	9	1	0.60 (0.13–2.78)	0.51
Folate supplementation						
No	111	58	317	57	1.0	0.74
Yes	79	42	239	43	0.94 (0.67–1.32)	0.74
Missing	416		1,079			
Folate Fortification						
No	300	50	740	45	1.0	
Yes	306	50	895	55	0.84 (0.70–1.02)	0.07
Water source						
Surface	118	19	304	19	1.0	
Ground	488	81	1,331	81	0.95 (0.75–1.20)	0.64
Municipal water						
Yes	245	40	609	37	1.0	
No	361	60	1,026	63	0.88 (0.72–1.06)	0.17
Nitrate exposure level						
<1 mg/L	127	21	353	22	1.0	
1–5.56 mg/L	351	58	931	57	1.02 (0.81–1.30)	0.68
>5.56 mg/L	127	21	351	21	0.97 (0.73–1.29)	0.71

Notes: * The values listed across from the variable names represent the *p*-values for the entire model. The *p*-values listed across from each category within variables represent the *p*-values for that category. ** 95% CI.

Table 4 summarizes the step-wise multivariable unconditional logistic regression model comparing cases to controls, without variables representing folic acid supplementation and pre-pregnancy weight, which were removed due to large numbers of missing data (68% and 21% respectively). After controlling for all other variables there is a trend-toward a modest increase in risk of congenital anomalies with exposure to nitrate concentrations greater than 1 mg/L, which is the local background nitrate concentration. This trend was present for both exposure categories 1–5.56 mg/L and >5.56 mg/L. However, this categorical nitrate variable does not attain statistical significance or show a dose-response relationship. 

**Table 4:** The progressive generation of the final multivariable logistic regression model describing the incidence of congenital anomalies by nitrate exposure level in a stepwise fashion such that potential confounding variables are added to the model one group at a time for all data from 1987–2006.

Variable	Basic Model: Matching Variables	Basic Model Plus Maternal Demographic Variables	Basic Model Plus Maternal Demographic and Health Variables	Basic Model Plus Maternal Demographic, Health and Water Source Variables	Final Model: Effect of Nitrate Exposure Level on Congenital Anomalies after Controlling for All Other Variables
Sex					
Female	1.0	1.0	1.0	1.0	1.0
Male	0.99 (0.82–1.20)	0.99 (0.82–1.21)	0.98 (0.81–1.20)	0.99 (0.81–1.20)	0.98 (0.81–1.20)
Season of conception					
Winter	1.0	1.0	1.0	1.0	1.0
Spring	1.04 (0.76–1.43)	1.05 (0.76–1.44)	1.05 (0.76–1.44)	1.05 (0.76–1.44)	1.05 (0.76–1.44)
Summer	0.97 (0.72–1.34)	0.99 (0.72–1.34)	1.00 (0.73–1.37)	1.00 (0.74–1.37)	1.00 (0.74–1.37)
Fall	0.97 (0.72–1.32)	0.96 (0.72–1.27)	0.97 (0.71–1.32)	0.97 (0.71–1.32)	0.98 (0.72–1.33)
Year of conception					
1987–1991	1.0	1.0	1.0	1.0	1.0
1992–1996	0.98 (0.71–1.36)	0.97 (0.70–1.36)	0.98 (0.71–1.37)	0.99 (0.71–1.38)	0.99 (0.71–1.38)
1997–2001	1.00 (0.71–1.36)	0.96 (0.72–1.27)	0.97 (0.73–1.29)	0.98 (0.74–1.31)	1.00 (0.75–1.34)
2002–2006	1.05 (0.73–1.51)	1.02 (0.70–1.45)	1.02 (0.71–1.48)	1.03 (0.72–1.49)	1.05 (0.72–1.52)
Maternal age					
<20		1.01 (0.71–1.44)	0.94 (0.66–1.35)	0.94 (0.66–1.35)	0.95 (0.66–1.36)
20–34		1.0	1.0	1.0	1.0
≥35		**1.38 (1.01–1.88)**	**1.40 (1.02–1.91)**	**1.39 (1.02–1.91)**	**1.40 (1.02–1.91)**
Parity					
0		1.0	1.0	1.0	1.0
1–2		**0.73 (0.60–0.90)**	**0.73 (0.59–0.89)**	**0.73 (0.59–0.89)**	**0.73 (0.60–0.90)**
3+		0.68 (0.43–1.07)	0.67 (0.43–1.06)	0.68 (0.43–1.07)	0.70 (0.43–1.06)
Smoker					
No			1.0	1.0	1.0
Yes			**1.28 (1.03–1.57)**	**1.28 (1.03–1.57)**	**1.27 (1.03–1.57)**
Diabetes					
No			1.0	1.0	1.0
Gestational			1.30 (0.70–2.41)	1.29 (0.70–2.40)	1.31 (0.71–2.44)
Other diabetes			0.97 (0.34–2.73)	0.97 (0.34–2.73)	0.96 (0.34–2.70)
Thyroid disease					
No			1.0	1.0	1.0
Yes			0.64 (0.14–3.00)	0.65 (0.14–3.05)	0.64 (0.14–3.01)
Water source					
Surface				1.0	1.0
Ground				0.85 (0.66–1.09)	0.70 (0.35–1.41)
Municipal water					
Yes				1.0	1.0
No				1.10 (0.81–1.51)	0.82 (0.63–1.07)
Nitrate exposure level					
<1 mg/L					1.0
1–5.56 mg/L					1.65 (0.83–3.27)
>5.56 mg/L					1.66 (0.81–3.42)

The data were then stratified by date of conception before or after 1 January 1998 (Table 5). For cases and controls conceived prior to 1998, there appeared to be a non-significant protective effect for exposure to drinking-water nitrate concentrations greater than 1mg/L. A trend toward reduced risk of congenital anomalies with nitrate exposure greater than the background concentration was shown for both nitrate exposure categories (1–5.56 mg/L and >5.56 mg/L.) For cases and controls conceived after 1998 there was a significantly increased risk of congenital anomalies with nitrate exposure from 1–5.56 mg/L and a trend toward an increased risk of congenital anomalies with nitrate exposure greater than 5.56 mg/L. For cases and controls selected after 1998, the effect size was slightly larger for the 1–5.56 mg/L than the >5.56 mg/L exposure level.

### 3.2. Discussion

This study builds on previous work to examine the association between drinking-water nitrate concentrations and incidence of major congenital anomalies in the agricultural region of Kings County, Nova Scotia, Canada by employing the use of province-wide population-based birth registry data which enabled controlling for factors that may have introduced confounding in previous studies and which allowed for inclusion of cases diagnosed *in-utero* with congenital anomalies in pregnancies that had been electively terminated before birth. This study also included users of both private wells and municipal water supplies in the study population and made use of county-wide groundwater nitrate seasonal surveys of concentrations and GIS methods to derive spatial estimates of individual-level nitrate exposure. 

**Table 5:** Final adjusted multivariate associations between incidence of congenital anomalies and all variables, including nitrate exposure level for cases and controls stratified by date of conception prior to or after 1998, when folic acid fortification was introduced to Nova Scotia.

Variable	Cases and Controls Conceived from 1987–1997 (n = 987)	Cases and Controls Conceived from 1998–2006 (n = 1,201)
Sex		
Female	1.0	1.0
Male	1.0 (0.75–1.34)	0.96 (0.73–1.24)
Season of conception		
Winter	1.0	1.0
Spring	1.0 (0.61–1.63)	1.12 (0.73–1.71)
Summer	1.01 (0.62–1.63)	1.01 (0.67–1.53)
Fall	0.98 (0.62–1.55)	0.98 (0.64–1.50)
Year of conception		
1987–1991	1.0	–
1992–1996	0.96 (0.69–1.35)	–
1997–2001	0.94 (0.62–1.42)	1.0
2002–2006	–	1.02 (0.75–1.39)
Maternal age		
<20	0.85 (0.50–1.43)	1.01 (0.61–1.68)
20–34	1.0	1.0
≥35	1.35 (0.76–2.39)	1.50 (1.03–2.19)
Parity		
0	1.0	1.0
1–2	0.77 (0.56–1.05)	**0.68 (0.52–0.90)**
3+	0.75 (0.39–1.45)	0.59 (0.31–1.11)
Smoker		
No	1.0	1.0
Yes	1.01 (0.74–1.38)	**1.55 (1.16–2.06)**
Thyroid disease		
No	1.0	1.0
Yes	1.55 (0.13–18.10)	0.36 (0.04–3.01)
Diabetes		
No	1.0	1.0
Gestational	1.63 (0.73–3.64)	2.27 (0.30–16.71)
Other diabetes	0.49 (0.06–4.15)	–
Water source		
Surface	1.0	1.0
Ground	2.50 (0.62–10.08)	0.54 (0.23–1.29)
Municipal		
Yes	1.0	1.0
No	0.78 (0.53–1.14)	0.82 (0.56–1.20)
Nitrate Exposure level		
<1 mg/L	1.0	1.0
1–5.56 mg/L	0.48 (0.10–1.60)	**2.44 (1.05–5.66)**
>5.56 mg/L	0.47 (0.11–1.90)	2.25 (0.92–5.52)

This study found that in univariable logistic regression analyses there was an increased risk of congenital anomalies with maternal smoking, and a protective effect with maternal parity of 1–2 (Table 3). Some previous studies have shown positive associations between smoking and congenital anomalies [29], though others have shown no association [30,31]. Previous studies have found that primiparity, especially in combination with older age and higher pre-pregnancy weight, is a significant risk factor for congenital anomalies [32,33]. Univariable analyses showed no effect of folic acid supplementation or fortification on incidence of congenital anomalies. Previous work in Nova Scotia has also shown that recommendations for folic acid supplementation had only a limited effect on incidence of neural tube defects, but that the incidence of neural tube defects was reduced after folic acid fortification [34]. It is likely that the onset of folic acid fortification showed no protective effect against congenital anomalies in this study because the analysis examined all major congenital anomalies, rather than exclusively neural tube defects, which are specifically associated with folic acid intake.

Using reliable outcome and covariate database, and a reasonably robust interpolated exposure variable, we were able to identify a trend (OR = 2.44) toward a positive association between congenital anomalies and drinking-water nitrate for the period 1998–2006, even while controlling for important cofounders (Table 5). When the study period as a whole is considered, there was a trend toward an increase in incidence of congenital anomalies with drinking-water nitrate exposure of 1–5.56 mg/L and >5.56 mg/L. When the data were stratified by conception before or after 1998, there was a non-significant protective effect for drinking-water nitrate exposure of 1–5.56 mg/L and >5.56 mg/L. This result was reversed for the cases and controls conceived after 1998, where there was a significant positive association between drinking-water nitrate levels from 1–5.56 mg/L and a non-significant positive association, though of slightly less magnitude, for drinking-water nitrate levels greater than 5.56 mg/L. 

The trend toward a positive association between congenital anomalies and drinking-water nitrate is consistent with previous studies. Dorsch *et al.* found that women who consumed water with total nitrate-nitrogen concentrations between 5 mg/L and 15 mg/L were 2.6 times more likely than women who consumed water with total nitrate-nitrogen concentrations below 5 mg/L to give birth to a child with a congenital anomaly (RR = 2.6, 95% CI = 1.6–4.1); those who consumed water with total nitrate-nitrogen concentrations above 15 mg/L experienced a 4.1 times greater risk of anomaly (RR = 4.1, 95% CI = 1.3–13.1) [11]. Arbuckle *et al.* observed that total nitrate-nitrogen concentrations of 26 mg/L showed a moderate increase in risk of CNS anomalies (ROR = 2.3; 95% CI = 0.73–7.29) when well water was considered in isolation [12]. Croen *et al.* (2001) found a progressively increased risk of anencephaly according to higher levels of total nitrate-nitrogen exposure for groundwater drinkers only (OR = 2.1; 95% CI = 1.1–4.0 for exposure concentrations of 5–15 mg/L; OR = 2.3; 95% CI = 1.1–4.5 for exposure concentrations of 16–35 mg/L; OR = 6.9; 95% CI = 1.9–24.9 for exposure concentrations of 36–67 mg/L) [13]. Cedergren *et al.* found that infants exposed *in-utero* to more than 2 mg/L nitrate-nitrogen had a marginally elevated risk of cardiac defects (OR = 1.18, 95% CI = 0.97–1.44) [14]. 

Two previous studies have shown no relationship between prenatal nitrate exposure and incidence of congenital anomalies, specifically neural tube defects and abdominal wall defects [35,36]. 

It is unclear why there appeared to be a protective association between drinking-water nitrate concentration and congenital anomalies before 1998 but an in increased risk with elevated drinking-water nitrate after 1998. There are several factors that may have contributed to this finding. The Nova Scotia Civic Address File was introduced in 1998, which likely improved the nitrate exposure classification of cases and controls from 1998 onward. Increased precision of addressing likely reduced random misclassification of nitrate exposure estimate and would have increased the power of the study to detect differences in incidence of congenital anomalies between drinking-water nitrate exposure groups. 

It is also possible that the reversal of the relationship between nitrate exposure and incidence of congenital anomalies for before and after 1998 is related to the fortification of grain products with folic acid in order to protect against neural tube defects. Our study examined all congenital anomalies, the majority of which were central nervous system anomalies, which includes neural tube defects. Both before and after 1998, congenital anomalies of the central nervous system were the most prominent type of congenital anomalies in Kings County. It is plausible that since prior to 1998, a large portion of the risk of congenital anomalies could be attributed to insufficient folic acid intake, and once this risk was abated after folic acid fortification the risk of congenital anomalies due to nitrate exposure was unmasked. 

Our study did not find evidence for a dose-response relationship between incidence of congenital anomalies and drinking-water nitrate exposure level; the odds ratio was higher for the lower exposure category (1–5.56 mg/L) than for the higher exposure category (>5.56 mg/L). This may be due to limited power to detect a difference at the higher exposure level, as there were only 21 cases and controls in this group. 

#### 3.2.1. Study Strengths

This study is unique because participants were selected from population-based databases that included data on elective second-trimester termination of pregnancies with antenatally diagnosed congenital anomalies, providing very complete case ascertainment. The wealth of high-quality information on maternal demographic and risk factors contained in the population-based databases also enabled controlling for a large number of maternal risk-factor for congenital anomalies, which enabled a more precise evaluation of the association between nitrates and congenital anomalies. 

Access to the Nova Scotia Civic Address file, which provided a precise latitude and longitude designation for all maternal addresses after 1998 also reduced likelihood of exposure misclassification due to false geographic location for the study period from 1998 onward. The robustness of the nitrate exposure variable was also enhanced by including users of both private wells and municipal water supplies in the study population and temporally monitored well data with fine geographic resolution using GIS.

#### 3.2.2. Study Limitations

The primary limitation of this study was that there were not sufficient cases for analyses to be sub-divided according to specific type of congenital anomalies. Environmental teratogens are expected to exert specific effects, contributing to the development of a relatively narrow range of congenital anomalies [37]. 

Another limitation of the study is that in rural areas the drinking-water nitrate exposure estimates were based on a study which took samples only in 1999 and 2000. Therefore, some water samples were taken after the birth of cases and controls. Too little data was available to reliably assess the temporal stability of drinking-water nitrate concentrations over the entire study period from 1987 to 2006. The study relied on linear mixed effects models to determine that sample location contributed to the most variation in drinking-water nitrate in Kings County over the study period. However, the trends in nitrate concentrations in Kings County found in our study are generally consistent with the existing literature. Previous work by Moerman and Briggins found that 13% of 237 wells in Kings County had nitrate concentrations greater than the MAC in 1989, which was similar to nitrate concentrations in Kings County in 1974 [38]. Work by Blair *et al.* using the same data at our study set also showed no monthly variation in nitrate concentrations. However, they did find that nitrate concentrations were higher in 1999 than in either 1989 or 2000 [39]. The work of both Moerman and Blair found elevated nitrate concentrations in Eastern Kings County bordering Canning and Port Williams, which is consistent with our nitrate exposure model [38,39].

The choice of ordinary kriging to model nitrate exposures in rural Kings County may have also limited the quality of the drinking-water nitrate exposure estimates. Kriging performs poorly when sample points are sparse [40], as is the case in the Western part of Kings County. Furthermore, our model was extrapolated to include all of Kings County, while all of the nitrate sampling points from both rural wells and municipal water supplies were located along the valley-floor. Outside the valley floor, nitrate concentrations were extrapolated. Overall, 72% of all civic address points in Kings County are in municipalities or in areas where the nitrate exposure model was represented by kriging. However, among Kings County residents using private wells to obtain drinking-water, 39% live outside the regions represented by kriging or municipal water supplies. Study participants living at these addresses had their nitrate exposure levels represented by an extrapolation of the kriging model, likely a poor representation of actual nitrate exposure. Therefore, there was likely some non-random exposure misclassification with more accurate nitrate exposure estimates made for those living along the valley floor than elsewhere in the study region.

Previous research in Kings County has found that those wells that have drinking-water nitrate levels that exceed the MAC are also more likely to contain fecal coliform bacteria and pesticides [23]. In general, a high nitrate concentration in a well is indicative of the additional presence of other contaminants. This study may be limited by residual confounding because the presence of other drinking-water contaminants that may be correlated with high nitrate concentrations were not evaluated. For example, pesticides exposure may be associated with increased incidence of congenital anomalies and is also positively correlated with nitrate concentrations [23,36].

## 4. Conclusions

This study builds on the existing research on drinking-water nitrate exposure and congenital anomalies by using a population-based, enhanced birth surveillance program for ascertainment of cases and controls and for controlling for important maternal and infant congenital anomaly-related risk factors. Over the entire study period there was a non-significant small increase in risk of congenital anomalies for drinking-water nitrate levels >1 mg/L. When only data from 1998 onward was considered, there was a significant increase in the incidence of congenital anomalies for drinking-water nitrate exposure levels of 1–5.56 mg/L compared to <1 mg/L. There was not a dose response relationship when nitrate levels >5.56 mg/L were considered, possibly due to inadequate power. The observed increase in the incidence of congenital anomalies with drinking-water nitrate exposure greater than 1 mg/L, which is just 10% of the Canadian MAC, is an intriguing finding and suggests that further investigation of the relationship between drinking-water nitrate and congenital anomalies at lower exposure levels is warranted. 

## List of Abbreviations

CNS: Central Nervous System
FAD: Fetal Anomaly Database
GIS: Geographic Information Systems
MAC: Maximum Allowable Concentration
NSAPD: Nova Scotia Atlee Perinatal Database

## Supplementary Files

Supplementary File 1 Supplementary Information (PDF, 112 KB)
