# Supplementary material for: A Population-Based Case-Control Study of Drinking-Water Nitrate and Congenital Anomalies Using Geographic Information Systems (GIS) to Develop Individual-Level Exposure Estimates
Source: Int J Environ Res Public Health. 2014 Feb 5;11(2):1803–23. doi: 10.3390/ijerph110201803 (PMC3945569; doi:10.3390/ijerph110201803)
Supplement: Supplementary File 1 — Supplementary Information (PDF, 112 KB) [file ijerph-11-01803-s001.pdf]

Supplementary Information

A Population-Based Case-Control Study of Drinking-Water Nitrate and Congenital Anomalies Using Geographic Information Systems (GIS) to Develop Individual-Level Exposure Estimates

---

Supplement: Interpolation and Extrapolation Methods

Prior to choosing the ordinary Kriging model used to estimate rural nitrate exposure, several models with variations in interpolation technique and parameters were created (Table S1). The model that had the mean error nearest to zero, as well as the mean standardized error closest to zero, the root mean square standardized error closest to one and the root mean square error closest to the average standard error was selected for use in the remaining analyses.

The selected model for the estimate of nitrate exposure in rural areas used ordinary kriging. The selected model for the estimate of nitrate exposure in rural areas used ordinary kriging. Kriging is a geostatistical means of estimating the value of an unknown point from a function of point-based weighted averages of known values at points in the neighbourhood. The resulting function represents a best linear unbiased estimate.

In our study we chose to use ordinary kriging with an exponential variogram and without a nugget effect. A minimum of 3 and a maximum of 5 neighbours were used to determine the nitrate value at each point. Neighbours were evenly selected from four quadrants (divided at 45°, 135°, 225° and 315°.) The division of quadrants and the selection of neighbours along the directions chosen were supported by the generally northeast (45°) flow of groundwater in Kings County. As all of the data points for the interpolation were located along the valley floor, the model was extrapolated to include all of Kings County.

This interpolation was validated by re-creating it using only 90% of the data points, and comparing it to the original interpolation.

Table S1. Prediction errors from various interpolation models of rural well nitrate concentrations (n = 1,113) from July 1999 to February 2000 in Kings County, Nova Scotia, and the associated model options [a,*].

**Interpolation model and parameterization**	**Prediction Errors**				
	**Mean**	**Root Mean Square**	**Average Standard**	**Mean Standardized**	**Root Mean Square Standardized**
	**Model Used for Analysis**				
Ordinary Kriging, Exponential variogram, 3–5 neighbours divided	0.02	7.59	7.71	0.01	1.08
	**Models Created for Comparison**				
Ordinary Kriging, Spherical variogram, 2–5 neighbours divided	0.80	8.73	8.81	0.08	1.29
Ordinary Kriging, Exponential variogram,2–5 neighbours divided	0.77	8.45	8.86	0.08	1.09
Ordinary Kriging, Spherical variogram, 5 neighbours divided	0.03	7.48	7.64	0.01	1.07
IDW. 5–10 neighbours	0.89	8.02			
IDW, 3–10 neighbours	0.89	8.02			
IDW, 2–5 neighbours	0.97	8.30			
IDW, 4–8 neighbours	0.88	8.10			
IDW, 4–8 neighbours, Search area divided	0.82	7.86			
IDW, 10–15 neighbours	0.94	7.98			
Ordinary Kriging, Exponential variogram, 2–5 neighbours, divided	0.05	7.64	7.84	0.01	1.08
Ordinary Kriging, Spherical variogram, 2–5 neighbours, divided	0.12	7.95	7.75	0.01	1.28

Notes: [a] IDW refers to Inverse Distance Weighting; * Average standard errors, mean standardized errors, and root mean square standardized errors are not generated when IDW is used for interpolation.

© 2014 by the authors; licensee MDPI, Basel, Switzerland. This article is an open access article distributed under the terms and conditions of the Creative Commons Attribution license (http://creativecommons.org/licenses/by/3.0/).
